# In vivo biopsy by laser confocal microscopy for evaluation of traumatic recurrent corneal erosion

**Published:** 2008-12-15

**Authors:** Tai-ichiro Chikama, Norihisa Takahashi, Makiko Wakuta, Naoyuki Morishige, Teruo Nishida

**Affiliations:** 1 Department of Ocular Pathophysiology, Yamaguchi University School of Medicine, Ube, Yamaguchi, Japan; 2 Department of Ophthalmology, Yamaguchi University Graduate School of Medicine, Ube, Yamaguchi, Japan

## Abstract

**Purpose:**

Laser in vivo confocal microscopy noninvasively provides images that are equivalent to high quality histology. We have now applied this technique to identify pathological characteristics of traumatic recurrent corneal erosion (RCE).

**Methods:**

Six eyes of six patients with traumatic RCE were studied. Corneas were examined with a slit lamp biomicroscope and with a laser in vivo confocal microscope (Heidelberg Retina Tomograph II–Rostock Cornea Module or HRTII-RCM) at various times after the onset of the most recent recurrence of corneal erosion.

**Results:**

Brightly reflective granular structures were detected by the HRTII-RCM system in the basal and wing cell layers of the corneal epithelium in all eyes affected by recurrent erosion. Activated keratocytes and scattered fine particles were also apparent in the shallow stroma of five of the six affected eyes. These features were not observed in the normal cornea.

**Conclusions:**

The HRTII-RCM system allows detection of characteristic abnormal structures in the cornea of individuals with traumatic RCE. The presence of granular structures in the corneal epithelium as well as persistent inflammation in the shallow stroma may contribute to the deterioration of the corneal epithelial cell alignment and to the weakening of adhesion between the basal epithelial cells and the basement membrane in RCE lesions.

## Introduction

Recurrent corneal erosion (RCE) is characterized by repeated episodes of corneal epithelial defects that are usually accompanied by the sudden onset of eye pain upon awakening as well as by hyperemia, photophobia, and tearing [1]. RCE is sometimes observed in individuals with diabetes mellitus or corneal dystrophies, but most patients with RCE have unilateral, localized lesions that are associated with an episode of mechanical trauma with shearing forces. RCE is thought to result from the loss of adhesion of basal corneal epithelial cells to Bowman’s layer [2]. The corneal epithelial adhesion complex maintains attachment of the epithelium to the underlying Bowman’s layer through the interaction of hemidesmosomes with type VII collagen. Histopathological analysis has revealed that the number of hemidesmosomes and the density of type VII collagen are reduced in RCE lesions associated with corneal dystrophies [3,4]. However, given the limited information available on RCE pathology, the precise mechanism of RCE development has remained unclear.

The development of confocal optics has allowed changes in corneal cells to be examined layer by layer [5,6]. Furthermore, the development of an in vivo laser confocal microscope with an attachment for the cornea (Heidelberg Retina Tomograph II–Rostock Cornea Module, or HRTII-RCM; Heidelberg Engineering, Heidelberg, Germany) has yielded new insight into corneal disorders by providing high resolution images of the cornea [7-9]. The HRTII relies on a 670 nm diode laser as the light source and yields images covering an area of 400 µm×400 µm with lateral digital resolution of 1 µm per pixel and digital depth resolution of 2 µm per pixel [10]. Confocal biomicroscopy has revealed deposits in basal corneal epithelial cells, subbasal microfolds, and streaks; damaged subbasal nerves; and altered morphology of the anterior stroma in patients with RCE [11]. The purpose of the present study was to characterize specific morphological features of the cornea in patients with traumatic RCE with the use of laser in vivo confocal microscopy to provide insight into the pathology of this condition.

## Methods

We undertook a retrospective evaluation of six patients with traumatic unilateral RCE at Yamaguchi University Hospital, Ube, Yamaguchi Japan. We also examined 30 volunteers (30 eyes) without any apparent pathology of the cornea. The six patients were referred to our hospital by their local ophthalmologists for diagnosis or treatment of their corneal epithelial disorders. All patients had at least two episodes of corneal erosion since the last corneal trauma. Sex, age, best corrected visual acuity (BCVA) at the initial visit, date of onset of the most recent erosion episode, and corneal lesion findings by slit lamp biomicroscopy and in vivo confocal microscopy were recorded. All subjects were informed of the aims of the study, and their consent was obtained. The study was approved by the institutional review board of Yamaguchi University Hospital.

In vivo confocal microscopy was performed with the HRTII-RCM system. Before examination, a drop of gel (Comfort Gel ophthalmic ointment; Bausch and Lomb, Berlin, Germany) was placed on the front side and inside of a TomoCap (Heidelberg Engineering GmbH, Heidelberg, Germany). One drop of topical anesthetic (0.4% Oxybuprocaine: Santen Pharmaceuticals, Osaka, Japan) and one drop of gel (Comfort Gel) were instilled into the lower conjunctival fornix of each eye. Corneal lesions were scanned by alternating vertical and horizontal movement of the applanating TomoCap from the upper edge of the lesion toward the lower edge. Several confocal microscopic images of tangential optical sections of the superficial, wing, and basal cell layers of the corneal epithelium, the stroma, and the endothelium were obtained for each eye. Oblique sections were also obtained when necessary. All in vivo confocal microscopy was performed by a single investigator (N.T.). No complications with the HRTII-RCM system were noted during examination.

## Results

### Clinical and slit lamp biomicroscopy findings

**Figure 1:**
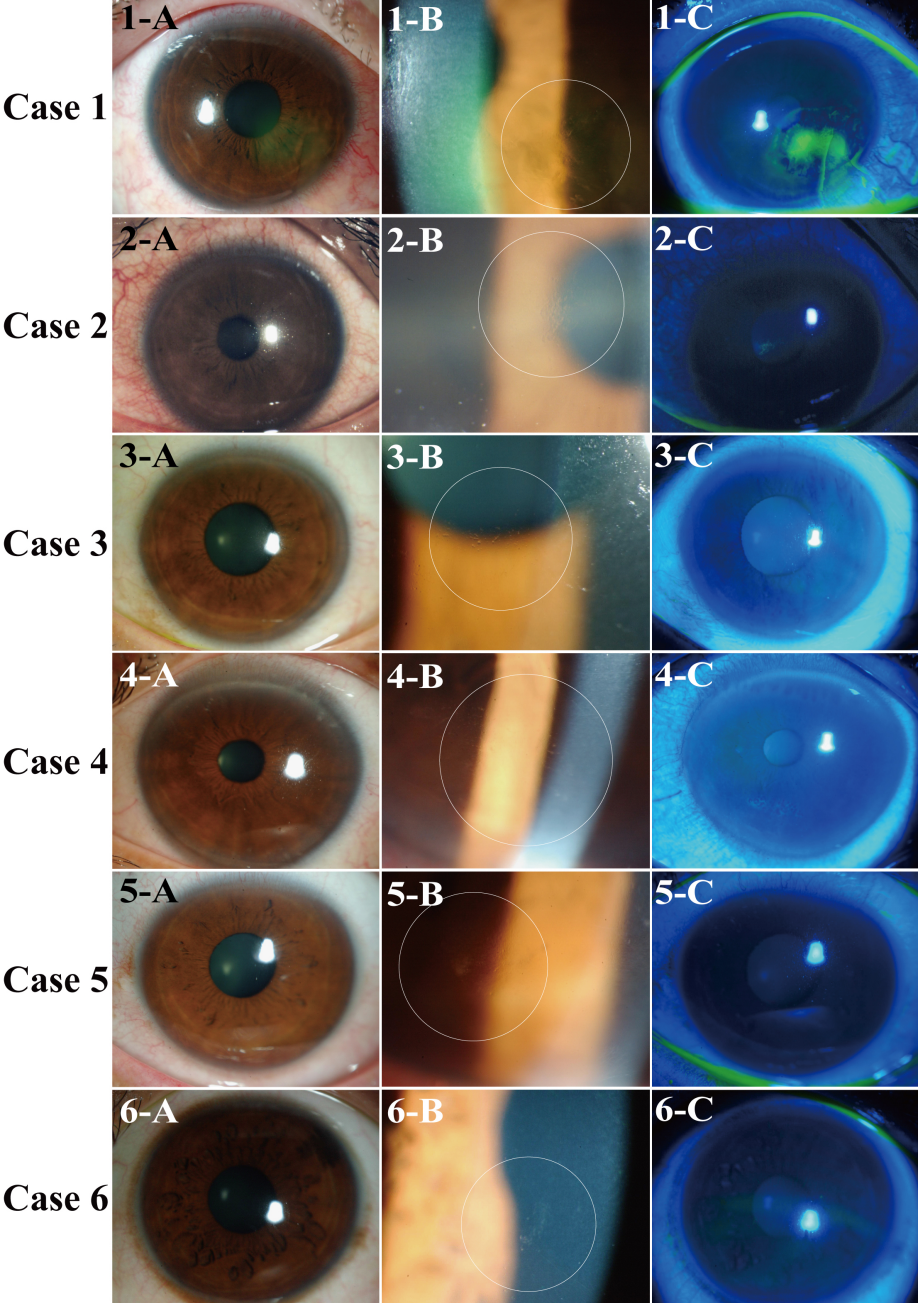
Slit lamp biomicroscopic findings of the six cases of traumatic RCE. The images of the first column (**A)** were obtained with diffuse illumination. The images of the second column (**B**) are higher power views obtained with a combination of retroillumination and proximal illumination. The images of the third column (**C**) show fluorescein staining. Semitransparent or small white deposits were observed in all cases (**B**). Corneal epithelial defects are stained green with fluorescein in cases 1 and 2 (**C**).

The clinical characteristics of the six patients with traumatic unilateral RCE are shown in Table 1. Four of the patients were men and two were women with an overall age of 37.8±9.6 years (mean±SD; range: 22−51 years). BCVA ranged between 0.7 and 1.5. No corneal changes characteristic of dystrophy of the corneal epithelial basement membrane such as map-dot patterns were observed by slit lamp biomicroscopy. Photographs of the corneas of all patients are shown in Figure 1. Corneal epithelial defects were detected by fluorescein staining in two patients (cases 1 and 2) within a few days of the most recent onset of RCE. Slit lamp examination revealed semitransparent or small white deposits aggregated at the site of recurrent erosion in the corneal epithelium of all six patients.

**Table 1:** Clinical characteristics of patients with traumatic RCE.

**Case**	**Age/sex**	**Eye**	**BCVA at initial visit**	**Symptoms at initial visit**	**Slit lamp findings**	**Number of RCE attacks**	**Time between erosion onset and HRTII-RCM examination (days)**	**Surgical treatment**
1	39/F	OD	0.7	Pain, tearing	Erosion, conjunctival hyperemia, semitransparent and small white deposits	2–3	2	Epithelial debridement
2	34/M	OS	1.2	Pain, foreign body sensation	Erosion, conjunctival hyperemia, semitransparent deposits	3–4	3	Epithelial debridement
3	51/F	OS	0.7	Pain	Conjunctival hyperemia, semitransparent deposits	3	10	None
4	42/M	OS	1.5	Diagnostic workup	Semitransparent deposits	4	16	Epithelial debridement
5	39/M	OS	1.2	Diagnostic workup	Semitransparent deposits	>10	30	None
6	22/M	OS	1.2	Diagnostic workup	Semitransparent and small white deposits	> 5	>30	Epithelial debridement

### In vivo laser confocal microscopy

**Figure 2:**
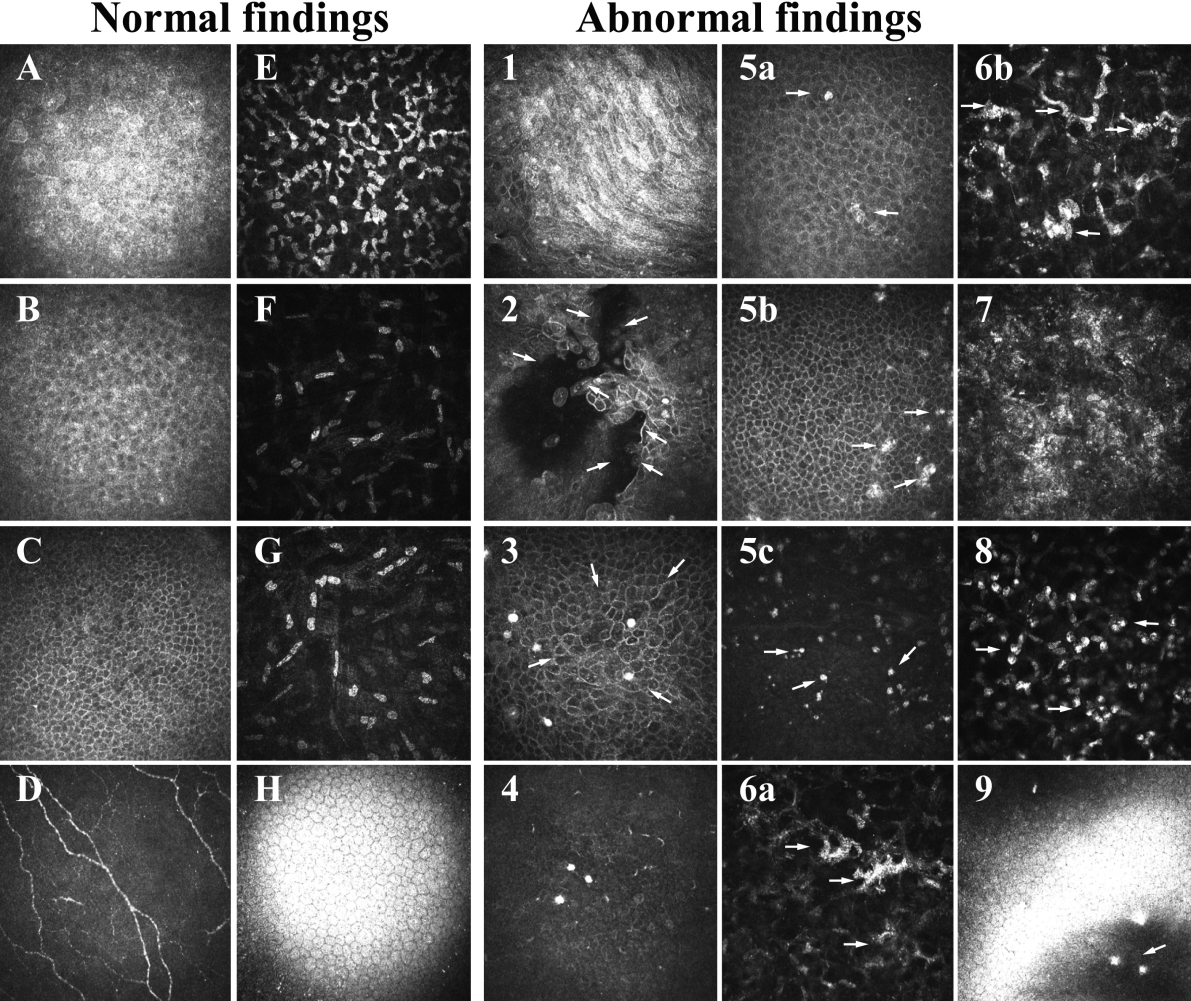
In vivo confocal microscopic images obtained with the HRTII-RCM system of all layers of the cornea in a normal eye and the six cases of traumatic RCE. The images of the first two columns represent areas of 400 µm×400 µm corresponding to the superficial epithelial cell layer (**A**), wing cell layer (**B**), basal cell layer (**C**), Bowman’s layer (**D**), shallow stroma (**E**), mid stroma (**F**), deep stroma (**G**), and endothelium (**H**) of the normal eye. Panel 1 (case 3) shows elongated and migrating superficial cells. Panel 2 (case 2) shows gaps (arrows) in the superficial epithelial layer due to epithelial defects. Panel 3 (case 3) shows the enlargement of basal epithelial cells (arrows). Panel 4 (case 2) shows the absence of subepithelial nerves as well as brightly reflective granular structures at the bottom of the basal cell layer and in Bowman’s layer. Panels 5a (case 4), 5b (case 6), and 5c (case 2) show brightly reflective granular structures (arrows) in the wing cell layer, in the basal cell layer, and in Bowman’s layer, respectively. Panels 6a (case 2) and 6b (case 5) show activated keratocytes (arrows) in the shallow stroma. Panel 7 (case 4) shows scattered fine particles in the shallow stroma. Panel 8 (case 1) shows infiltrated cells, likely neutrophils (arrows), in the mid stroma. Panel 9 (case 2) shows a small number of keratoprecipitates (arrow) on the endothelium.

Abnormal findings by in vivo laser confocal microscopy in the corneas affected by RCE (Figure 2) included (1) irregularity in the alignment of superficial epithelial cells; (2) gaps in the epithelial cell layers; (3) enlargement of the basal epithelial cells; (4) the absence or a reduced number of subepithelial nerves; (5) brightly reflective granular structures in the basal and wing cell layers of the epithelium and in Bowman’s layer; (6) activated keratocytes in the shallow stroma; (7) scattered fine particles in the shallow stroma; (8) infiltration of inflammatory cells in the mid stroma; and (9) keratoprecipitates on the corneal endothelium. None of these findings were detected in the 30 normal eyes examined.

We divided the six cases into three groups on the basis of the time between the onset of the most recent episode of RCE and examination by in vivo confocal microscopy. Cases 1 and 2 were in the early stage group because in vivo confocal microscopy was performed within two and three days after the onset of RCE, respectively. All of the abnormal findings for RCE with the exception of keratoprecipitates for case 1 were observed with the HRTII-RCM system in the corneas of these two cases. Cases 3 and 4 were in the mid-stage group because in vivo confocal microscopy was performed at 10 and 16 days, respectively, after the onset of RCE. The in vivo confocal microscopic images for cases 3 and 4 revealed enlarged basal cells, brightly reflective granular structures in the corneal epithelium and Bowman’s layer, activated keratocytes in the shallow stroma, and scattered fine particles in the shallow stroma. In contrast, gaps in the epithelial cell layers and inflammatory cell infiltration in the mid stroma were not observed in this group. Cases 5 and 6 constituted the late stage group in which in vivo confocal microscopy was performed 30 days or more after the onset of RCE, respectively. Both corneas of this group manifested brightly reflective granular structures in the corneal epithelium and Bowman’s layer whereas case 5 also exhibited activated keratocytes in the shallow stroma and scattered fine particles in the shallow stroma. The in vivo confocal microscopic findings for all cases are summarized in Table 2. They reveal that brightly reflective granular structures were detected with the HRTII-RCM system not only in the basal cell layer but also in the wing cell layer of the corneal epithelium and in Bowman’s layer in all eyes affected by traumatic RCE. Furthermore, both scattered fine particles, and activated keratocytes were apparent in the shallow stroma of the lesion in five of the six cases.

**Table 2:** Summary of in vivo confocal microscopic findings in patients with traumatic RCE.

**Finding**	**Normal**	**Case**						**Frequency in RCE cases**
		**1**	**2**	**3**	**4**	**5**	**6**	
Irregular epithelial cell alignment	-	+	+	+	-	-	-	3/6
Gaps in epithelial cell layers	-	+	+	-	-	-	-	2/6
Enlarged basal epithelial cells	-	+	+	+	+	-	-	4/6
Absence or reduced number of sub-basal nerves	-	+	+	-	+	-	-	3/6
Brightly reflective granular structures in the epithelium and Bowman’s layer	-	+	+	+	+	+	+	6/6
Activated keratocytes in the shallow stroma	-	+	+	+	+	+	-	5/6
Scattered fine particles in the shallow stroma	-	+	+	+	+	+	-	5/6
Cell infiltration in the mid stroma	-	+	+	-	-	-	-	2/6
Keratoprecipitates on the endothelium	-	-	+	+	-	-	-	2/6

-: absence, +: presence, on the respective finding with the confocal microscopy. The abnormal findings shown in Figure 2 were not detected in the normal eyes.

**Figure 3:**
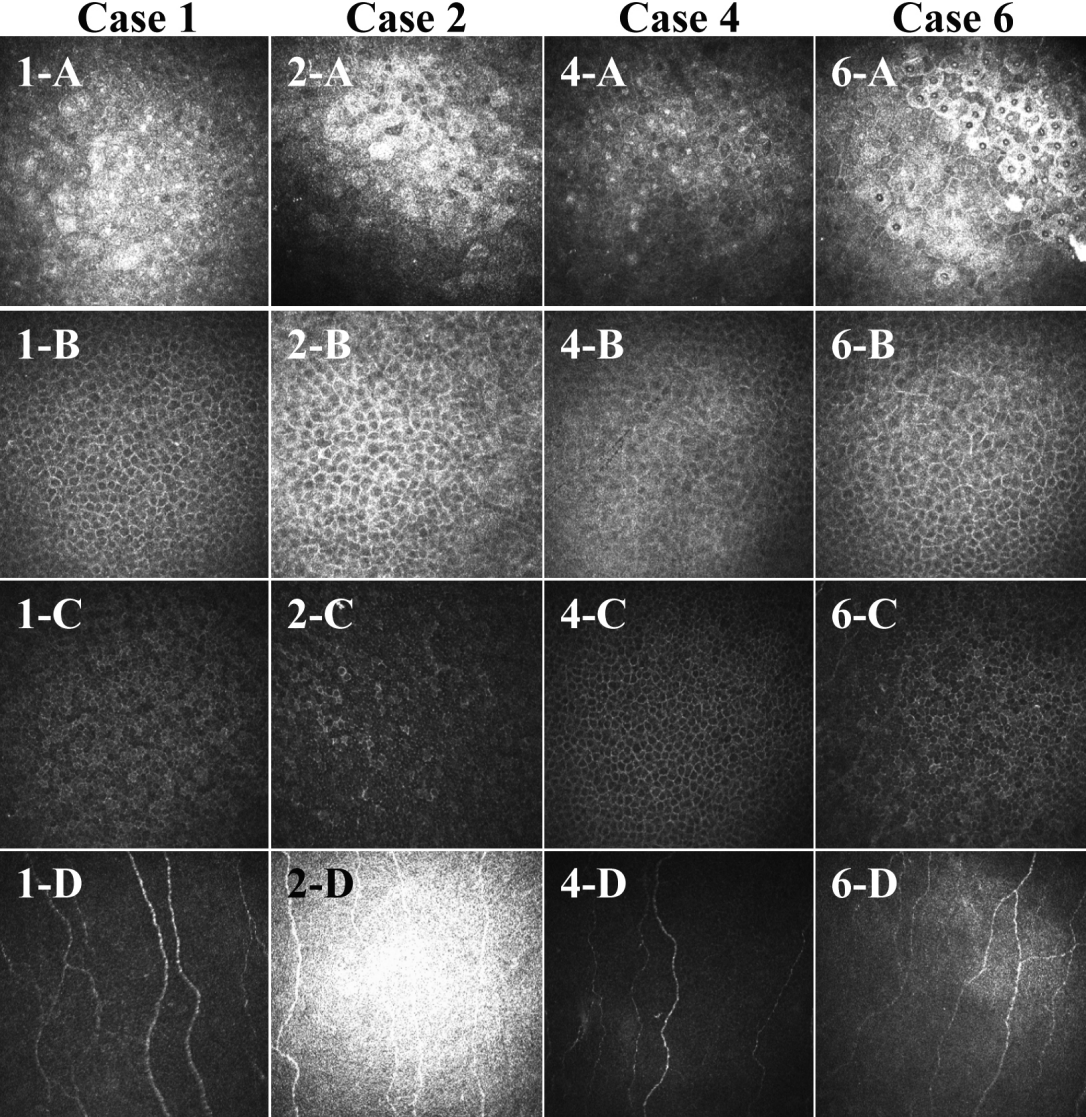
In vivo confocal microscopic images for four patients with traumatic RCE after corneal epithelial debridement of the affected area. The images represent the superficial epithelial cell layer (**A**), wing cell layer (**B**), basal cell layer (**C**), and Bowman’s layer (**D**). No brightly reflective granular structures were apparent in any layer of the four corneas one month after treatment.

Corneal epithelial debridement was performed for the affected area in four patients (cases 1, 2, 4, and 6). These four patients had not developed any recurrence of erosion, and no brightly reflective granular structures in the corneal epithelium were detected with the HRTII-RCM system one month after the treatment (Figure 3).

## Discussion

We have identified candidate pathological characteristics of the lesions associated with traumatic RCE with the use of in vivo laser confocal microscopy. Among these characteristics, the irregularity of superficial epithelial cell alignment is suggestive of the active migration of epithelial cells to cover epithelial defects whereas the gaps in epithelial cell layers appear to reflect such defects or incomplete epithelial cell migration. The enlargement of basal epithelial cells likely represents a compensatory response to the lack of overlying epithelial cells. The absence or reduced number of subepithelial nerves suggests that the nerves are destroyed or damaged in association with the epithelial defect. Given that subepithelial nerves appeared normal in cases 5 and 6, it is possible that the damaged nerves regenerate with time after epithelial erosion. The infiltration of inflammatory cells in the mid stroma and the presence of keratoprecipitates on the endothelium are indicative of an acute inflammatory response secondary to epithelial defects. These characteristics were apparent in the early and mid stages of the erosion episodes and are likely not specific to RCE. In contrast, the brightly reflective granular structures in the corneal epithelium and Bowman’s layer were observed at all stages of RCE in the cases studied. These structures likely represent deposition of hyperreflective material, most probably cell debris. In vivo confocal microscopy performed after LASIK has also revealed brightly reflective particles at the corneal interface [10,12,13]. These particles have been suggested to be metal particles from the blade of the microkeratome, sponge particles, powder from surgical gloves, ocular surface debris such as lipid products, or implanted corneal epithelial cells [12,13]. In the case of traumatic RCE, it is possible that foreign material such as ocular surface debris is deposited at the time of the injury and persists in the corneal epithelial layer. The presence of activated keratocytes, which are characterized by reflective nuclei and visible cytoplasmic processes, and scattered fine particles in the shallow stroma is suggestive of a chronic inflammatory response. Inflammation due to epithelial defects would be expected to result in the activation of keratocytes, which produce matrix metalloproteinases and contribute to haze induction [14,15].

The cause of RCE after traumatic injury is thought to be the failure to recover tight adhesion between the wounded corneal epithelial cells and the underlying stroma. RCE can also result from loss of adhesion due to a defect in the basement membrane in the cornea of individuals with epithelial basement membrane dystrophies [16]. In such cases of map-dot-fingerprint dystrophy, Meesmann’s dystrophy, or Reis-Buckler’s dystrophy, confocal microscopy has revealed several pathological characteristics including deposits in basal epithelial cells and cysts as well as subbasal microfolds and streaks [11,17]. The HRTII-RCM system has recently revealed an abnormal basement membrane protruding toward the epithelium as well as epithelial microcysts in cases of map-dot-fingerprint dystrophy [9]. None of the patients of the present study was diagnosed with epithelial basement membrane dystrophy on the basis of these previous findings in the epithelium and basement membrane with the HRTII-RCM system or slit lamp examination.

Even in asymptomatic stages of traumatic RCE as exemplified by cases 4, 5, and 6, we observed brightly reflective granular structures in the basal and wing cell layers of the corneal epithelium as well as a persistent mild inflammatory response in the shallow stroma. Although RCE can occur in patients with epithelial basement membrane dystrophies and after traumatic injury, its pathology appears to differ between the two types of case. Even though the present study includes only a small number of cases, our findings provide a basis for speculation on a possible mechanism for traumatic RCE. This condition may thus result from a failure of reconstruction of cell-cell or cell-matrix adhesion structures at the lesion site. This failure may contribute to deterioration of the corneal epithelial cell alignment and to the weakening of adhesion between basal cells and the basement membrane. It may also lead to mild inflammation involving the activation of keratocytes in the shallow stroma and the consequent operation of a vicious cycle involving the epithelium and shallow stroma.

The HRTII-RCM system allows detection of abnormal structures in the cornea. Indeed, in vivo laser confocal microscopy noninvasively provides high resolution images that are equivalent to high quality histology, having been termed “in vivo biopsy.” A longitudinal study of patients with RCE is required to provide insight into the natural course of the pathological changes associated with this condition. Furthermore, the efficacy of treatments for RCE such as debridement and bandage contact lenses warrants evaluation with the HRTII-RCM system.

In conclusion, in vivo laser confocal microscopy reveals pathological characteristics of RCE. Although the diagnosis of RCE should be made on the basis of clinical progress and careful examination with a slit lamp biomicroscope, the in vivo laser confocal microscope is a powerful tool that can contribute to the diagnosis and management of RCE.
